# Optimization of a closed rat tibial fracture model

**DOI:** 10.1186/s40634-018-0128-6

**Published:** 2018-05-02

**Authors:** Kareem Obayes Handool, Sahar Mohammed Ibrahim, Ubedullah Kaka, Muhammad Aarif Omar, Jalila Abu, Md Sabri Mohd Yusoff, Loqman Mohamad Yusof

**Affiliations:** 1 Department of Companion Animal Medicine and Surgery, Faculty of Veterinary Medicine, Universiti Putra Malaysia, UPM, 43400 Serdang, Selangor Malaysia; 2 Department of Preclinical Sciences, Faculty of Veterinary Medicine, Universiti Putra Malaysia, UPM, 43400 Serdang, Selangor Malaysia; 3 Department of Veterinary Clinical Studies, Faculty of Veterinary Medicine, Universiti Putra Malaysia, UPM, 43400 Serdang, Selangor Malaysia; 4 Department of Veterinary Pathology & Microbiology, Faculty of Veterinary Medicine, Universiti Putra Malaysia, UPM, 43400 Serdang, Selangor Malaysia

**Keywords:** Fracture model, Rats, Tibia, In vivo

## Abstract

**Background:**

The use of a closed fracture model has become the preferred model to study the fracture healing process, given that the periosteum and the soft tissue surrounding the fracture site play an important role in the fracture healing process. Some techniques like osteotomy, drilling the long bones and the use of the guillotine-like apparatus to induce fracture are characterized by some undesirable effects and complications. The aim of this study is to optimize and evaluate an in vivo fracture model using three-point bending pliers that can be used to study secondary bone fracture healing in rats.

**Methods:**

Modified three-point bending pliers were used as a device to create the closed rat tibial bone fracture that was prefixed with an intramedullary pin (23 G × 1^1^/_2_″) in rats. The exact location of the induced closed fracture was along the long bone. The presence of bone comminution, and the fracture bone alignment were immediately examined after the induction of the fracture until the 6th week.

**Results:**

All fractures induced were transverse, located in the middle to proximal one third of the tibia, and they all healed without complications. Bone union as shown radiographically occurred within 2–3 weeks postoperative. The average angle of the fracture line with the axis of the tibia was 89.41 ± 2.11°. The lateral and anterio-posterior pin angulation views were 167.33 ± 3.67° and 161.60 ± 4.87° respectively. The average length of proximal end of the fractured bone in comparison with the whole length of intact bone was 41.02 ± 3.27%. There was a significant difference in percentage of the gross callus area and gross callus index, while there was no significant difference in X-ray callus index. There was no significant difference of the gross callus area between slight comminution (*n* = 4) and non comminution (*n* = 21).

**Conclusion:**

The optimized rat tibial fracture model resulted in mainly transverse tibial mid-shaft fractures with minimal bone comminution and absence of surrounding soft tissue damage. The size area of consequent soft callus formation and the extent to which the closed fracture model was reproducible are very good outcomes making it feasible for in vivo laboratory research use.

## Background

The large number of seriously injured patients requiring treatment and the long-term morbidity associated with skeletal injury inevitably results in greater socioeconomic impact. Hence, to improve the quality of patient care and reduce the costs of treatment, there are ongoing research and clinical works to develop novel therapies to enhance bone fracture healing that will shorten the recovery period (Yukata et al., 2014; Zhang et al., 2017). The process of fracture healing starts following the disruption of vessels that causes hematoma and activates thrombotic factors in the coagulation cascade. This leads to an inflammatory response within which cytokines and growth factors recruit osteoprogenitor and mesenchymal cells that generate a granulation tissue at the edges of the fractured bone. The granulation tissue is then replaced by bone, formed either by intramembranous (direct) bone formation or through a hyaline cartilage tissue that is subsequently mineralized to woven bone through endochondral (indirect) bone formation (Shapiro, 2008; Ghiasi et al., 2017). This then results in the fracture being stabilized and the tissue is later remodeled. Manually inflicted fractures of the rat tibia with either internal or external fixation have been described by several authors as a model to study fracture healing. The inadequate stability of internal or external fixation leads to variable degrees of displacement and repeated movement of the bone fragments during the healing period (Slätis and Rokkanen, 1965, 1967; Glatt et al., 2017). The ideal experimental fracture should be of consistent site, with the standard degree of bone damage, soft tissue injury, stability and displacement of the bone fragments, and the time needed for bone union should also be standardized (Greiff, 1978). The soft tissues also have an important role in fracture bone healing. For example, callus formation around the fracture provides temporary stabilization. The release of various mediators and sprouting of newly formed blood vessels are also contributed by the soft tissues in the milieu. In addition, the surrounding muscles contribute to stabilization and strengthening of the long bone fracture (Otto et al.*,* 1995; Loi et al., 2016).

In 1984, Bonnarens and Einhorn described a fracture model in the rat based on a previously designed model by Jackson et al. (1970). In this model, osteosynthesis was performed before the fracture using an intramedullary (IM) pin inserted through the knee in a retrograde fashion (Aurégan et al., 2013). Then, a fixed weight dropped onto the bone placed on a blunt guillotine created a standardized closed mid-shaft fracture of the femur.

Some studies have reported recurrent complications, including death, misplaced fracture, excess comminution and deep infection in a rat model of femoral fracture using this blunt guillotine technique (Aurégan et al., 2013; Haffner-Luntzer et al., 2016).

Moreover, it appears that fractured bone comminution in various degrees has been particularly difficult to control during fracture healing (Pei and Fu, 2011; Ghiasi et al., 2017). As the degree of fracture comminution can affect the formation of callus, there was association between the rat fracture model used with the resultant comminution and the soft callus produced. Using similar principles, Grieff (1978) used a guillotine device to create bone fractures in rat tibiae fracture models and the result showed minor comminution but high displacement of bone fragment and pin angulation of more than 10°. Otto et al. (1995) who used three-point pliers in the rat tibial fracture model demonstrated minimal edema and death, but a high percentage of shortening of the fractured tibiae with the callus being still visible until six months post-surgery and pin angulation of 80 ± 2°. Owing to these limitations of the existing fracture models, there is a need for a reliable fracture model of the laboratory animals especially in rats that is feasible and reproducible. The aim of the study is to optimize a closed rat tibial fracture model based on works of Grieff (1978) and Otto et al. (1995) and to evaluate the extent to which the model can overcome the current limitations and offer a better technique that creates a rat fracture model with more consistent and reproducible results.

## Methods

### Animals

All the experimental designs in this study were approved by the Institute Animal Care and Use Committee (IACUC) of University Putra Malaysia (UPM/IACUC/AUP-R028/2015). The preliminary study used ten (*n* = 10) and seventy one (*n* = 71) rat cadavers of 30 weeks and 8 weeks old respectively. The cadaver rats were humanely killed for other experiments following the guidelines of the AICUC. The fracture was induced using the three-point bending pliers as described below. In the in vivo study, a total of twenty five (*n* = 25), 8 weeks old female Sprague-Dawley rats with an average weight of 187.04 ± 10.15 g were used. An acclimatization period of one week was allowed before the experiment started**.** The rats were starved for 12 h prior to surgery following the acclimatization period.

### Surgical procedure

All surgeries were performed on the animals under general anaesthesia using a premixed combination of Ketamine Hydrochloride (Narketan®, Troy Laboratories PTY Limited, Australia) and Xylazine (Ilium Xylazil-100®, Troy Laboratories PTY Limited, Australia) (McKelvey and Hollingshead, 2004). Ketamine (40 mg/kg) and Xylazine (5 mg/kg) was given by intramuscular injection to each of the rats for surgery and radiography. Xylocaine 2% local anaesthesia (Xylocaine®, AstraZeneca, UK) was also given subcutaneously. Routine surgical aseptic skin preparation of the surgical site was performed.

The surgical site was approached through 4 mm skin incision craniolaterally of the stifle joint using a scalpel blade # 15 (BlBraun®, Bbraun, Germany). The tibial plateau was exposed with the stainless steel and a sharp probe was introduced manually through the tibial plateau, cranial to the attachment of the anterior cruciate ligament and between the anterior horns of both menisci to determine the area of intramedullary pin entrance. A 23 G 1^1^/_2_″, needle (BlBraun®, Bbraun, Germany) was inserted till it met resistance at the distal end of tibial bone. The depth of penetration of the needle was approximately 22 mm. After the intramedullary pin being introduced completely through the tibia, the excess proximal needle end was cut flush using bone cutter. The incision was closed with simple interrupted non-absorbable 4/0 nylon suture (Ethilon*, Ethilon. LLC)**.**

**Fig. 1:**
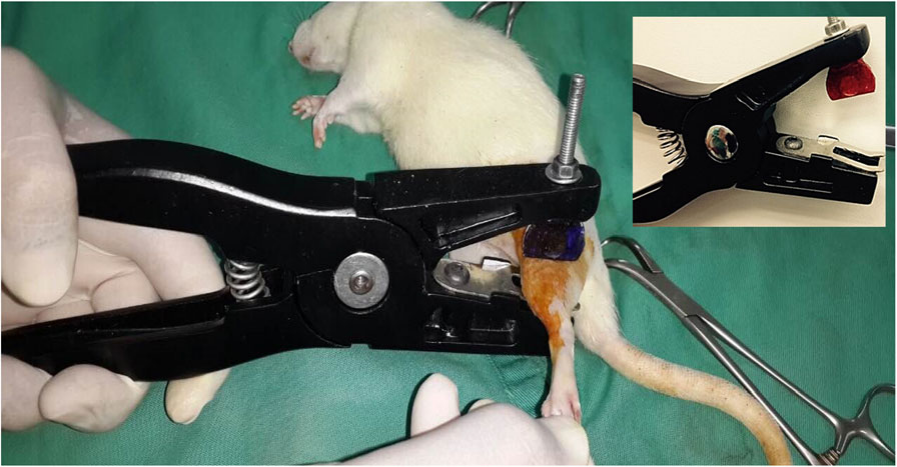
The photograph shows the application of the device to create tibial fracture in a rat (inset, the modified three-point bending pliers used)

To induce the fracture on the rat tibia, specially modified three-point bending pliers (Schippers®, MS, Netherlands) (Fig. 1), was constructed by modifying the upper circle compressed jaw of the Otto et al. (1995) device according to the compressor blade of Grieff, (1978) a device with dimensions of edge 10 X 0.1 mm. In addition, the two support jaws of the three-point bending pliers were modified to 7 mm instead of 17 mm as in the Otto et al. (1995) device.

The pliers were positioned with the two-support jaw on the medial side of the tibial mid-shaft and the pressing jaw on the lateral side. After application of the pliers the position was controlled visually and the pliers were closed till a cracking sound was heard and the pliers’ resistance suddenly falls. The pressure was immediately released thereafter. The same principles mentioned above were applied to create tibial or femoral fracture in rat cadavers. The fracture was created on the left tibia and the counter lateral right intact tibia was utilized as the control. Each animal was kept in individual cages to allow free ambulation post-surgery. A dose of 5 mg/kg Tramadol (Analab®, Biolab, Thailand) was given once a day for 3 days as analgesia following the surgery. Gross observation and radiological evaluation were done at different time points following euthanasia using a high anaesthetic dose of pentobarbital at 20 mg/kg IP injected (Society and States, 2013).

### Gross evaluation of the fracture

The skin of the fractured leg was inspected for any wound caused by the pliers’ jaws. As it was a closed fracture with intramedullary pin pre-fixed into the fractured bone, the fracture was considered stable and no additional post-operative external supportive bandage was required. The dissected tibiae were collected from both the induced- facture and non-fracture limb (control). Five (*n* = 5) rats were sacrificed at each time point, namely at 1st, 2nd, 3rd, 4th, and 6th week post-surgery. Gross callus index and callus area size were determined from the dissected fracture tibiae and the control (Greiff, 1978; Otto et al., 1995; Eastaugh-Waring et al., 2009; Aurégan et al., 2013). A dissecting stereo-microscope (HUVITZ; HSZ-645TR, Korea) fitted to a 3.1 M pixel digital camera (VIS Imaging UC3010, Malaysia) (millimeter unit of measurement) was used to capture dissected bone images and measure the callus width (mm) of the rat left tibial fracture healing with callus formed. The contralateral rat right tibia was used as control and the data was used to determine the ratio for the callus index using ImageJ software (1.48 V, NIH, USA). The same software was used to measure the callus area size, callus index, pin angulation, proximal fracture, whole length fracture and normal control bones.

### Radiological evaluation of the fracture

Immediately after surgery and every week thereafter, the fractured and contralateral intact tibiae were radiographed on two views (anterioposterior (AP) and lateral views) using an X-ray machine (Orange 10040HF, Econet Medical, Korea). The obtained radiographs were used for objective evaluation of the bone healing using criteria such as bone union, size of callus formation, degree of bone comminution, pin angulation, and radiograph callus index. The radiograph callus Index is defined as the maximum width of the callus formation of the mid-tibia fracture, including the original bone divided by the width of the contralateral control bone. A comminuted fracture is a splinter or break of the bone into more than two fragments which radiographically appeared at the fracture site. Three types of comminution were to be observed: firstly, no comminution (no third fragment) is detected on the two radiological observation views of anterioposterior and lateral; secondly, slight comminution with smaller fragments where the size is smaller than the pin diameter, i.e. < 1.1 mm, and thirdly, severe comminution (≥ one large fragment larger than the diameter of the intramedullary pin) (Aurégan et al., 2013).

### Histological examination of the fracture

The animals were sacrificed at week 1, 2, 3, 4, and 6 post fracture and samples of the dissected tibiae were collected (*n* = 5) from the fractured limb at each week. The bone was cut 2 mm proximally and distally to the fracture site (where gross callus formation was evident), fixed in formaldehyde 10% for 24 h, decalcified in 10% formic acid for 4 days before being further processed, sectioned (5 μm) and stained with hematoxylin and eosin (H&E) using standard procedures (Kiernan, 2000; Bancroft and Gamble, 2007).

### Statistical analysis

The statistical SPSS Software version 20 (Armonk®, IBM, USA) and SAS Software (SAS®, TAKS, England) were used for the data analyses. We used 2-paired samples student’s *t*-test to compare the group without comminution and the group with slight comminution, and one way ANOVA for other data analysis. Standard errors of mean were reported with *P < 0.05* were considered to be statistically significant.

## Results

The initial study using 30 weeks old rat cadavers (*n* = 10), showed that the tibial fracture produced was predominantly comminuted, oblique and distally located. In the next experiment using 8 weeks old rat cadavers (*n* = 65), the result showed that non comminution, transverse and mid-shaft of the tibia were the predominant fractures (Table 1).

**Table 1:** Comparison of types of fracture created in rat tibiae at two different ages

Assessment parameters	Transverse	Oblique	Non-comminuted	Comminuted	Mid- shaft	Distal
30 weeks	2	8^A^	1	9^A^	3	7^A^
8 weeks	62^B^	3	61^B^	4	60^B^	5

^A^superscript indicates the highest number in 30-week-old rats, while ^B^ superscript indicates the highest number in 8 weeks old rats

**Fig. 2:**
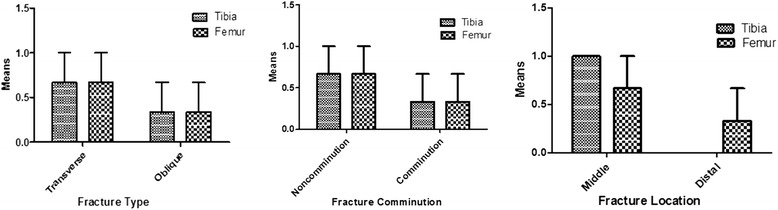
Tibial and femoral fractures created in rat cadavers using modified three-point bending pliers. Data were expressed in means ± SE and error bars denote the standard error

In the other experiment, 8 weeks old rat cadavers (n = 6) were used to compare the type of fractures induced between femoral and tibial bones. The type of fracture of femur and tibia appeared to be mainly transverse and non-comminuted fractures and located at mid-shaft of the bones (Fig. 2). Data comparison between the tibia (*n* = 3) and femur (n = 3) showed no significant difference between the two groups for the comminution fracture type and location (Two-tailed *t-*Test).

In the in vivo study, all induced fractures were found to be in the middle third of the tibiae. The mean operation time was 3.90 ± 0.55 min. All the animals survived the surgery without any complications, soft tissue damage at the fracture site or stiffness of the adjacent knee and ankle joints. No wound or necrosis and signs of severe edema were seen at the fracture site. Twenty four hours after the fracture, the rats showed normal behavior, started walking around on three legs and lifting the fractured leg. One to two weeks post-surgery, the rats started to bear weight on the affected limb. All the rats started to show weight bearing on all four limbs after 2 weeks onwards.

**Fig. 3:**
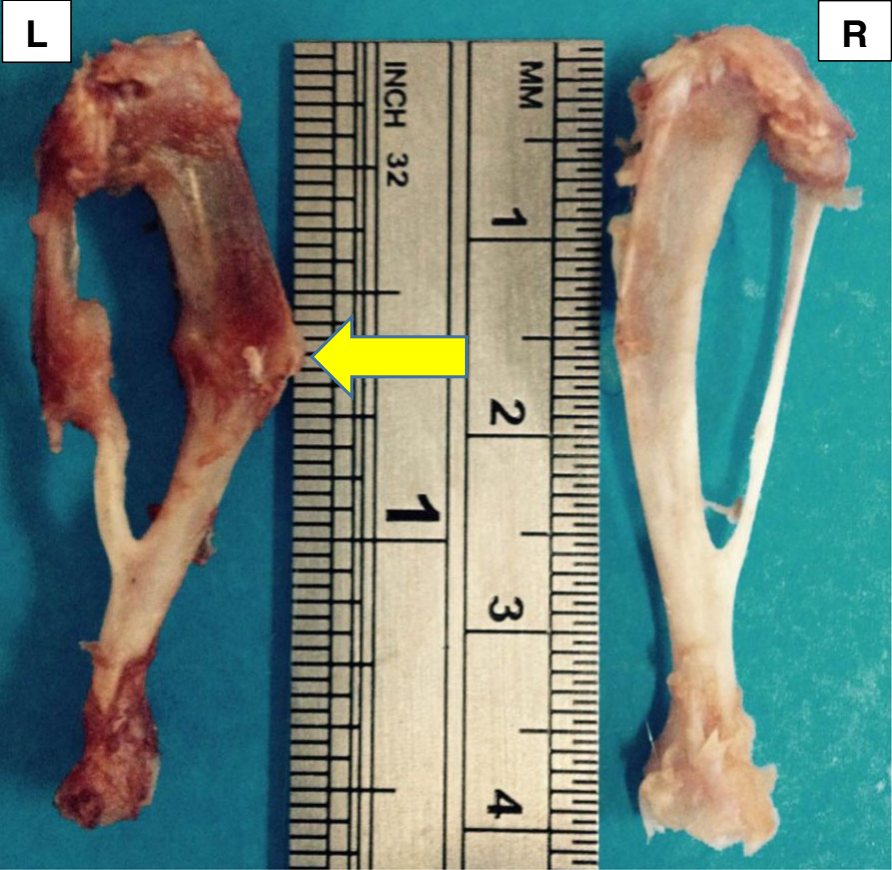
The callus formation of fractured tibia (arrow) with intramedullary pinning at the left (L) (yellow arrow) and intact tibia at the right (R) for comparison

The gross appearance of the callus area after euthanasia and tibial dissection showed clear callus formation at the left tibia in comparison of the right tibia (control) (Fig. 3).

**Fig. 4:**
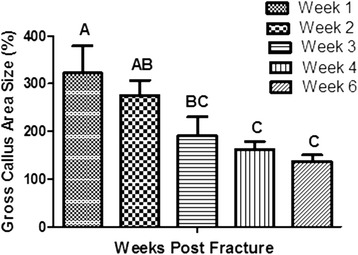
Bar chart of gross callus area percentage. The chart shows means ± SE in different weeks (*n* = 25) post rat tibial fracture. Error bars denote the standard error. Means in each column with different superscripts were significantly different

**Fig. 5:**
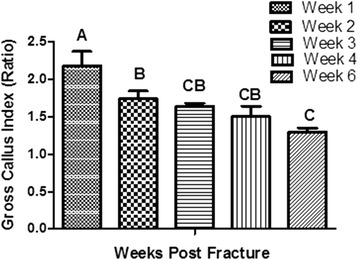
Graph of the gross callus index formation. The chart shows weeks 1, 2, 3, 4 and 6 following surgery (n = 25). Error bars denote the standard error. Means in each column with different superscripts are significantly different (*P < 0.0006*)

Dissected callus formation was observed at week 1 and decreased gradually right through week 6. Statistical analysis of gross callus area (Fig. 4) and gross callus index (Fig. 5) generally showed significant differences at week 1 in comparison with the other weeks (*P < 0.0065;* One-way ANOVA)**.**

Radiological evaluation of the type of fracture was determined by measurement of the angle between the fracture line and the long axis of the tibia. The main type of fracture induced was transverse (89.41° ± 2.11). The average length of proximal end fractured bone to the whole length of intact bone was 41.02 ± 3.27%. The mean angulation of the IM pin from the lateral and anterioposterior view positions were 167.33° ± 3.67° and 161.47 ± 4.87° respectively.

**Fig. 6:**
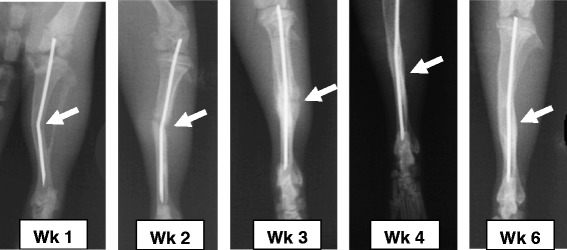
Radiographs of rat tibial fractures at different weeks post fracture. The radiographs show stages of bone fusion during fracture healing (arrows). The bone union was barely visible at week 1 and 2, slightly clear in week 3, showed good clarity at week 4 and was completed in week 6

Callus formation surrounding the fracture site became clearly visible on the radiographs starting from the second week. In the third to fourth weeks, the callus increased in density and the fracture line could hardly be visualized. At the fourth week onwards, more of the fracture line disappeared and the fractured bone was fused completely (Fig. 6).

**Fig. 7:**
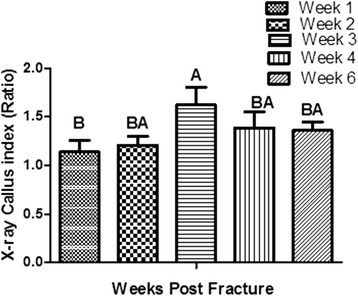
Bar chart of the radiograph callus indices. The graph shows weeks 1, 2, 3, 4 and 6 following surgery (n = 25). Error bars denote the standard error of means. Means with different letters were significantly different

The radiograph callus index was barely measurable at week 1, very small amounts to hardly being seen in week 2, clear and reached the highest point in weeks 3 before decreasing gradually in weeks 4 and 6. The means ± SE of the radiograph callus indices at 1, 2, 3, 4 and 6 weeks post-operatively were 1.14 ± 0.12, 1.21 ± 0.09, 1.72 ± 0.19, 1.95 ± 0.16 and 1.35 ± 0.09 respectively. However, statistical analysis of the radiograph callus index showed no significant differences among different weeks (One-way ANOVA) (Fig. 7).

The slight comminuted fracture was observed in four animals and the other was without any bone comminution. For bone comminution evaluation of all tibial fractures (*n* = 25), only four (16%) showed slightly comminuted fractures while the other twenty one (84%) were non-comminuted. However, in comparing the callus formation between the two groups, there was no significant alteration observed between the slight comminuted fracture group (*n* = 4) and the non-comminuted group (*n* = 21) with mean percentage callus area size being 0.81 ± 0.64% and 0.52 ± 0.32% respectively (*P > 0.444*; Two samples of *t*-Test). Table 2 summarizes the comparison between types of fracture that were induced in rat tibia.

**Table 2:** Comparison of types of induced tibial fracture and fracture healing in rats (n = 25)

	Slight Comminuted	Non- comminuted	*P-value*
Type of fracture (n)	4	21	
Animal weight (g)	176.75 ± 7.50	189 ± 28.39	*0.106*
Surgery procedure time (mins)	3.5 ± 1.0	3.97 ± 1.98	*0.496*
Callus area %	0.81 ± 0.64	0.52 ± 0.32	*0.444*
1-Anteroposterior (AP)	161.03 ± 5.42	161.56 ± 15.48	*0.905*
2- Lateral (Lat.)	165.03 ± 7.68	167.77 ± 8.96	*0.552*

Slight comminuted and non-comminuted fractures have the same values (2-tail *t*-Test). Data are expressed in means ± SE and important at *P < 0.05*

**Fig. 8:**
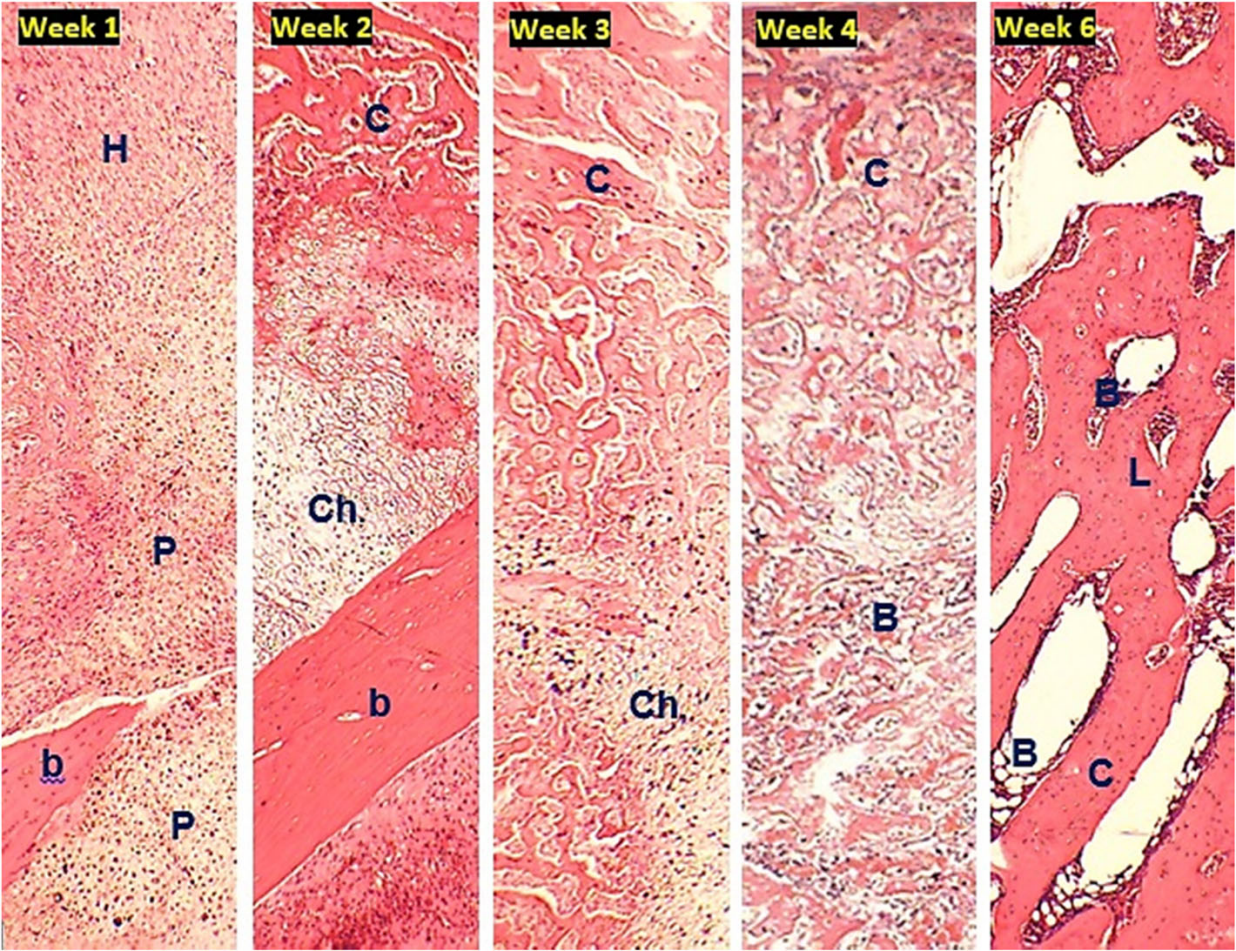
Photomicrograph of fracture healing for five weeks showing the hematoma (H), normal bone (b) and proliferative chondrocytes (P) in week 1, chondrocytes (Ch.), normal bone (b) and cancellous bone (C) in week 2, hypertrophic chondrocytes (Ch.) and cancellous bone (C) in week 3, cancellous bone (C) and bone marrow (B) in week 4 and cancellous(C) and lamellar bone (L) with bone marrow (B) in week 6

Histological examination of the fractured bone showed hematoma and chondrocytes at proliferative stage at week one post fracture, while at week two the chondrocytes zone and the cancellous bone increased. At week three and four the cancellous bone increased with appearance of hypertrophic chondrocytes and bone marrow. The fracture healing was ultimately observed at week six where the cancellous and lamellar bone together with bone marrow was predominantly present with the disappearance of chondrocytes (Fig. 8).

## Discussion

The use of a closed fracture model is considered the choice model for studying the fracture healing process. This is important because the periosteum and the soft tissue surrounding the fracture site play an important role during the fracture healing processes (Davis et al., 2015). Osteotomy of the tibia or femur or making a drill in the rat bone are the most common bone healing models investigated by orthopedic scientists (Otto et al., 1995). A guillotine-like apparatus described by Bonnarens and Einhorn (1984) was characterized by some disadvantages, including frequent reports of comminution, varying rates of complication and bending. However, the angulation in this study was only measured in the lateral plane. Aurégan et al. (2013) used a rat model of femoral fracture and in this model, a fixed weight was dropped to the bone placed on a blunt guillotine to create closed mid shaft fractures. The bone was prefixed with an IM pin before the fracture. The results show that 12% of the fractures created were not suitable for the research because of death, deep infection or a non-acceptable fracture, while the remaining fractures displayed slight comminution (63%).

Jackson et al. (1970) used a pneumatic punch press to produce closed femoral fractures in rats. In this technique model, 15% of fractures were excluded due to the fact that they were not at the right location and not transverse. Hulth (1989) used manual force to produce a closed fracture model of the rat tibia but it was difficult to control or reproduce in a standard manner, making the method unsuitable as a model. Other reported fracture models use crushing by a pair of pliers to produce a closed tibia fracture (Newman et al., 1985). It was associated with serious damage to the surrounding soft tissues.

Having stated a number of the setbacks and limitations of the earlier fracture model techniques, the current modified device used in this study takes into account those issues and the importance of soft tissue surrounding the fracture. Our current modified three point bending pliers used to create the closed bone fractures was constructed by modifying the upper circle compressed jaw of the Otto et al. (1995) device according to the compressor blade of the Grieff, (1978) device with dimensions of 10 mm X 1 mm (Fig. 1). In addition, the gap distance of the two support jaws of the three-point bending pliers was reduced to 7 mm instead of 17 mm as in the Otto et al. (1995) device. Using this modified device resulted in a fracture without any significant damage or effects on the surrounding soft tissue. The device was easy to use and control, and the IM pin prefixed before the fracture served to stabilize the bone fragments and enables the animals to immediately regain their ambulatory function. Even though the prefixed IM could not provide rigid stabilization to the fractured bone, it could minimize the bone comminution during the fracture and at the same time could resolve the problem of post fracture limb shortening and rotation of bone fragments (Omerovic et al., 2015) in rats.

The technique also produced consistent outcomes with minimal complications such as skin abrasion and laceration at the fracture site. This is contrary to the results of other previous studies of this nature that reported recurrent complications, misplaced fractures and excess comminution with deep infection in this rat model (Jackson et al. 1970; Newman et al., 1985; Hulth, 1989; Aurégan et al., 2013).

This current study reported no significant bending of the intramedullary 23 G needle used between the lateral and anterioposterior (AP) radiographic planes. On the other hand, Bonnarens and Einhorn (1984) in a related study, reported significant differences between AP and lateral angulation. The significant bending observed in the AP plane angulation in the Bonnarens and Einhorn (1984) study could be explained by the more lateral impact of the blunt guillotine on the limb (Aurégan et al., 2013) in comparison to the technique used in the present study.

In another study using goats, a closed tibial fracture was created and stabilized with a cast and at two weeks the callus volume was found to reach its maximum (Den Boer et al., 1998). However, in our current study there was a significant increase in the gross callus area in week one compared to the subsequent weeks. This difference may be as a result of differences in animal species and mode of fixation.

Eastaugh-Waring et al. (2009) showed that both fracture stiffness and callus indices have been reported to increase postoperatively with time. This could be considered as a consistent feature regardless of fixation method used (Eastaugh-Waring et al., 2009). However, our result showed that the callus indices decreased over 6 weeks as the fracture became more stable toward the later phase of fracture healing.

The gross callus area and gross callus index of the rat fractures in this study generally showed an initial increase at week 1 and gradually decreased towards week 6. However, a slightly different trend was shown for the radiograph callus indices, which showed the highest value at weeks 3 and 4. This could be because of the fact of the difference in the time onset of soft and hard callus formations. In this study, hard callus formation was more evident on the radiograph at 3 or 4 weeks after the fracture.

Our result also showed that the radiograph callus index was lower at week 2 and reached a maximum level at weeks 3 and 4 before decreasing at week 6. This finding was different from the progressive increase observed in an experimental osteotomy conducted in dogs stabilized with an external fixator, where the amount of callus increased over time until week six (Markel, 1990). The variation may likely be attributed to the different species and fixation method used in both experiments.

We found that there was non-significant difference in percentage of the gross callus area size produced between slight comminuted and non-comminuted fracture bone. This suggested that a slight comminuted fracture created using the current modified device gave minimal influence at the early phase of the fracture healing. In contrast, Aurégan (2013) found a significant difference in the amount of callus produced between slight comminuted and non-comminuted fractures in rats.

Fracture healing is completed within six to eight weeks post induction of injury (Sfeir et al., 2005). Temporary characteristics of secondary bone healing involve six stages that can be identified. These include an initial stage during which hematoma is formed in addition to inflammation. This is followed by angiogenesis and cartilage formation which leads to the stage of the calcification of the cartilage. The fourth stage is the stage of cartilage removal which results in bone formation and the final stage which is ultimately bone remodelling (Oryan et al., 2015). In agreement with those normal stages of fracture healing, the present result showed the callus formation at the fracture site was observed after the first three weeks, followed by gradual increase in bone mineralization and ultimately the complete bone union and stabilization at week six post fracture.

## Conclusions

The modified three-point bending pliers used in this present study are practical to create a reproducible closed fracture in rats with very minimal post-operative complications. It was also found to be a relatively simple device that can be used to prepare in vivo fracture models particularly of secondary fracture healing.
